# Supplementary material for: In vivo biomechanical responses of neonatal brachial plexus when subjected to stretch
Source: PLoS One. 2023 Aug 30;18(8):e0290718. doi: 10.1371/journal.pone.0290718 (PMC10468090; doi:10.1371/journal.pone.0290718)
Supplement: S1 File — (PDF) [file pone.0290718.s001.pdf]

N1 [n=16]	MSC	N2 [n=10]	Median	N3 [n=8]	Ulnar
Load (N)	Strain (%)	Load (N)	Strain (%)	Load (N)	Strain (%)
8.8	35.5	24.2	28.5	15.5	33.1
5.9	35.6	23.5	24.0	15.1	37.3
7.2	26.8	19.5	34.3	16.2	27.9
9.2	41.5	16.8	23.7	15.4	34.3
7.9	28.9	16.2	30.5	15.5	35.0
7.6	33.7	12.3	26.8	10.1	29.2
7.7	24.3	27.2	39.5	15.1	32.8
9.1	37.5	15.3	33.5	16.0	33.9
5.6	27.8	19.8	30.0		
7.0	48.6	21.1	72.5		
6.5	25.3				
12.0	55.9				
12.0	52.4				
6.4	39.8				
4.3	48.9				
8.0	24.8				

N4 [n=13] Load (N)	Radial Strain (%)
25.4	26.1
27.3	23.0
29.6	54.3
21.5	41.1
20.7	43.4
16.4	39.1
37.9	41.7
33.7	46.9
44.7	38.5
17.2	21.5
16.4	21.2
31.6	51.4
	54.4
