# Supplementary material for: In vivo biomechanical responses of neonatal brachial plexus when subjected to stretch
Source: PLoS One. 2023 Aug 30;18(8):e0290718. doi: 10.1371/journal.pone.0290718 (PMC10468090; doi:10.1371/journal.pone.0290718)
Supplement: Attachment — Submitted filename: Review BBPI.docx [file pone.0290718.s002.docx]

It is a good study with a few concerns

1. BP and MSC is not standard abbreviation so avoid using it.
2. Line no 69 to 71 Only one study quoted which is by the same author which does not rule out bias. Please explain.
3. Line 227 correct the spelling from chord to cord.
4. Line 266 again study by the same author is quoted ,need to justify.
5. Line 277 the logic seems correct that surrounding tissue may allow to have much load stress but need to prove further.
6. Did author studied about sensory and motor nerve threshold with stretch and load, If so is there any difference in sensory and motor load stress?
7. Clinical utility of the study need to emphasize. Specially how to imply the finding of study in routine practice to reduce the incident of BBPI.
8. Although a very good article suggest that even after increase in the **Cesarean section ,the reduction in BBPI is not significant. Vaginal deliver may not be the only cause of it.**

(Semin Perinatol 2006 Oct;30(5):276-87. doi: 10.1053/j.semperi.2006.07.009.

Cesarean section on request at 39 weeks: impact on shoulder dystocia, fetal trauma, neonatal encephalopathy, and intrauterine fetal demise

[Gary D V Hankins](https://pubmed.ncbi.nlm.nih.gov/?term=Hankins+GD&cauthor_id=17011400)  1 , [Shannon M Clark](https://pubmed.ncbi.nlm.nih.gov/?term=Clark+SM&cauthor_id=17011400), [Mary B Munn](https://pubmed.ncbi.nlm.nih.gov/?term=Munn+MB&cauthor_id=17011400)
