# Supplementary material for: In vivo biomechanical responses of neonatal brachial plexus when subjected to stretch
Source: PLoS One. 2023 Aug 30;18(8):e0290718. doi: 10.1371/journal.pone.0290718 (PMC10468090; doi:10.1371/journal.pone.0290718)
Supplement: Attachment — Submitted filename: Editorial comments.docx [file pone.0290718.s003.docx]

**EDITORIAL COMMENT**

Overall, this study is clinically quite relevant and innovative. This study is unique because most of the earlier studies have been conducted on adult cadavers, while this study has been conducted on neonatal animal piglet models.
Material & methodology – is well explained
Results - The results of this study can be quite helpful to design prevention strategies so as to minimize the incidence of neonatal brachial plexus injuries.
Discussion – Authors have described an association between the failure mechanisms among the various BP levels to different kinds of brachial plexus injury and its relation with brachial plexus anatomy.

But still there are some interesting queries raised by reviewers, which need to be looked into. I am attaching the reviewer’s comments which have been suggested. Authors are required to make the required amendments in their study, before we proceed with publication.

**Decision – ACCEPTED AFTER MAJOR REVISION**

**Please fine attached the reviewer comments below:**

**REVIEWER 1**

Very concise and good study.

The study is clinically very relevant.

There are minor grammatical errors but those are not relevant.

Having piglets for the study presents with less discrepancy as compared to with humans - **Accept**

**REVIEWER 2**

Research study is very unique and conducted keenly and excellently.

Material and methods are explained in detail.

Results are well explained. S

tatistical analysis done correctly. I **strongly recommend** this article

**REVIEWER 3**

Strong points- Novel study, in-vivo biomechanical properties of neonatal brachial plexus was studied.
Neonatal piglet animal model was chosen, very much similar to human Brachial plexus.
Very extensive technology has been used to study bilateral brachial plexus
Extensive set up with an actuator, a load cell, a clamp & 3-D imaging system has been used to study the biomechanical properties at 3-4 points in BP.
Time & effort has been put up in the study to locate the various failure points along the entire length of brachial plexus.
Maximium load was calculated & also strain at maximum load was also noted.
Histology of stretched tissue was performed to show changes in the tissue.

Appropriate statistical analysis has been done, Graphs has been made to show the results obtained.
May have clinical correlation in decreasing the overall incidence of stretching of NBPP

Weak points- Very stringent conditions are required to perform such extensive studies.
This type of study requires a longer period of time.
Suggestions - Authors can add clinical correlation of various parameters measured in the discussion section.
One flowchart depicting various steps in methodology section
Authors can explain clinical implication of measuring maximum load & biomechanical properties of different nerves - **Accept**

**Reviewer 4**

Need to use standard abbreviation eg: BP (brachial plexus is not used commonly), MSC need to specify at first place. It is a good study with a few concerns

1. BP and MSC is not standard abbreviation so avoid using it.
2. Line no 69 to 71 Only one study quoted which is by the same author which does not rule out bias. Please explain.
3. Line 227 correct the spelling from chord to cord.
4. Line 266 again study by the same author is quoted, need to justify.
5. Line 277 the logic seems correct that surrounding tissue may allow to have much load stress but need to prove further

6. Did author study about sensory and motor nerve threshold with stretch and load, If so is there any difference in sensory and motor load stress?
7. Clinical utility of the study need to emphasize. Specially how to imply the finding of study in routine practice to reduce the incident of NBPP.
8. Although a very good article suggest that even after increase in the Cesarean section, the reduction in NBPP is not significant. Vaginal deliver may not be the only cause of it.(Semin Perinatol 2006 Oct;30(5):276-87. doi: 10.1053/j.semperi.2006.07.009.
Cesarean section on request at 39 weeks: impact on shoulder dystocia, fetal trauma, neonatal encephalopathy, and intrauterine fetal demise Gary D V Hankins  1 , Shannon M Clark, Mary B Munn - **Accepted after Major Revision**
